# Probable European-profile *Borrelia*-associated myocarditis in an Australian patient with immune resolution following early therapy: a case report

**DOI:** 10.3389/fmed.2026.1784007

**Published:** 2026-04-15

**Authors:** Katerina Mitsakos, Pamela E. Heuer, Tiziana Beninati, Daniel J. Polley, Richard J. Schloeffel, Bernard J. Hudson

**Affiliations:** 1 Division of Medicine, Department of Infectious Diseases, Royal North Shore Hospital, Northern Sydney Local Health District, Sydney, NSW, Australia; 2 Faculty of Medicine and Health, The University of Sydney, Sydney, NSW, Australia; 3 Eve Technologies Corporation, Calgary, AB, Canada; 4 Royal North Shore Hospital, Northern Sydney Local Health District, Sydney, NSW, Australia; 5 Department of Microbiology, NSW Health Pathology, Royal North Shore Hospital, Sydney, NSW, Australia

**Keywords:** antimicrobial therapy, Australia, borreliosis, immune signatures, Lyme-like illness, myocarditis, serology, tick-borne disease

## Abstract

Tick-associated illnesses are increasingly recognised in Australia, yet the epidemiology and clinical manifestations of *Borrelia burgdorferi sensu lato* remain uncertain in the absence of confirmed local isolates and reliance on diagnostics validated for European and North American strains. These limitations complicate interpretation and may contribute to delayed recognition of systemic manifestations, with potential progression to Debilitating Symptom Complexes Attributed to Ticks (DSCATT). We describe a case consistent with European-profile *Borrelia* infection presenting with early inflammatory myocarditis evolving to mild cardiomyopathy and complete clinical and immunologic recovery after antimicrobial therapy. The patient had recent travel to tick-endemic regions of Scandinavia (Denmark and Sweden) in mid-July 2022 and subsequently developed symptoms following reported tick exposure at North Head, Sydney. Symptom onset occurred on 16 August 2022. The first serology, which was reactive, was obtained 13 weeks after symptom onset and demonstrated broad IgG reactivity (VlsE variants, OspC, p58, p39), fulfilling CDC two-tier and EUCALB immunoblot criteria for disseminated *Borrelia* infection. Whole-blood multiplex PCR was negative. Treatment comprised 28 days of intravenous ceftriaxone followed by 12 weeks of doxycycline. Immune-mediator profiling identified an early pro-inflammatory signature (IL-6, TNF-*α*, IFN-*γ*) with concurrent lymphoid activation (TNF-*β*) and prominent endothelial activation (fractalkine), followed by a dominant reparative vascular profile characterised by PDGF-AA, EGF, and sCD40L. Clinical indices improved substantially, with Horowitz Questionnaire Score decreasing from 47 to 18 and Karnofsky Performance Status increasing from 70 to 100 by Month 12. Serologic contraction and immune normalisation paralleled clinical recovery. Immune-cell kinetics aligned with cytokine-defined cluster transitions (B1/B3 inflammatory activation to B2/H vascular-repair programming), with early redistribution and reduced circulating B cells and NK cells followed by recovery to low-normal ranges by 12–24 months. At the systemic level, leukocyte counts showed early leukocytosis, mid-course suppression during stromal–vascular repair, and complete normalisation, consistent with immune containment without persistent inflammation. The mature serologic and immunologic profile does not distinguish between infection acquired during European travel or subsequent Sydney exposure, and local transmission cannot be inferred. This case illustrates probable *Borrelia*-associated inflammatory cardiomyopathy with full resolution and highlights the value of integrated serologic and immune-signature profiling in complex tick-associated presentations.

## Introduction

Tick-borne diseases (TBD) are increasingly recognised in Australia, with rickettsial notifications rising (1, 2). The epidemiology and clinical spectrum of *Borrelia burgdorferi sensu lato*–associated illness remain uncertain, and no human isolate has been confirmed locally (3, 4). *Borrelia*-like organisms have been identified in Australian ticks, reflecting ecological diversity without established human pathogenicity (5).

In the absence of region-specific diagnostics, delayed recognition may contribute to Debilitating Symptom Complexes Attributed to Ticks (DSCATT) and other post-infectious syndromes recognised in Australian clinical guidance (6–10). Profiling soluble immune mediators provides a framework for distinguishing persistent inflammation from post-infectious immune resolution (11).

Myocarditis represents a rare manifestation of Lyme borreliosis (LB), occurring within the broader spectrum of Lyme carditis. Cardiac involvement is reported in approximately 4–10% of untreated cases and 0.2–4% in contemporary surveillance cohorts (12–20). Most presentations involve atrioventricular (AV) conduction block, and differentiation from viral or vaccine-associated myocarditis requires exclusion of SARS-CoV-2 infection and vaccine-related inflammation (21–23). International guidelines emphasise early cardiac evaluation in suspected LB (13, 14, 16, 18).

We describe a probable *Borrelia burgdorferi s.l.* infection with a European-pattern immunoblot profile, presenting with inflammatory cardiomyopathy in Sydney, and demonstrating full clinical and immunologic recovery after early sequential antimicrobial therapy (AMT).

This case illustrates how integrated serologic and immune-signature evaluation can guide diagnosis and disease status in an Australian setting.

## Case presentation

### Clinical course

On 15 August 2022, a 62-year-old man (179 cm, 82 kg; BMI 25.6) walked in tick-endemic bushland at North Head, Sydney. The following day, he developed arthralgias and myalgias.

Symptom onset (SO) on 16 August 2022 serves as the clinical reference point (Clinical Day 0 of illness).

Past medical history included psoriasis, intermittent arthropathy, inactive for 30 years, and hypercholesterolaemia. There was no prior history of cardiovascular disease and no family history of premature cardiovascular disease, autoimmune rheumatic disease, or cardiomyopathy. A prior (2000) coronary artery calcium score (CACS) was 0.

In mid-July 2022, approximately 4 weeks before SO, he had travelled to *Borrelia*-endemic regions in Northern Europe, including Denmark, Bornholm Island, and Sweden; no tick bite was recalled. He had previously lived in the United Kingdom and travelled globally including the United States.

Six days before SO, he received mRNA COVID-19 booster. Vaccine-associated myocarditis was considered unlikely given persistently normal troponin and stable cardiac imaging (10, 21–23).

On 19 August 2022 (Day 3 after onset), he noted diffuse lateral thigh erythema and a discrete 10 cm medial erythematous patch. No tick was observed. SARS-CoV-2 PCR was negative. Empirical therapy for cellulitis (cefalexin) commenced but discontinued due to gastrointestinal intolerance.

The full chronological clinical timeline, including symptom onset, serologic milestones, and treatment phases, is summarised in Supplementary Table S1b.

### Cardiac investigations

Between September and October 2022, the patient developed progressive exertional tachycardia, fatigue, and bilateral jaw pain exacerbated by exertion and mastication. Electrocardiography (ECG) demonstrated a new left bundle-branch block (LBBB).

Wearable smartwatch data suggested persistent tachycardia. Holter monitoring (24 October 2022) showed sinus rhythm (59–120 bpm; mean 87) with rare atrial ectopy and no ventricular arrhythmia.

Computed tomography pulmonary angiography (CTPA) on 11 November 2022 excluded pulmonary embolism.

Transthoracic echocardiography (TTE) on 16 November 2022 demonstrated mild global dilated cardiomyopathy without significant valvular pathology.

High-sensitivity troponin measured on 21 October and 18 November 2022 remained <2 ng/L. CK ranged from 51 to 43 U/L. CRP measured 20.3 mg/L on 18 November 2022.

Coronary CT angiography on 21 November 2022 confirmed normal coronary arteries (calcium score 0).

The first laboratory evidence of *Borrelia* exposure was obtained on 18 November 2022 (SO +13 weeks), demonstrating IgG reactivity already established, by ELISA and confirmatory immunoblot. Hence it was not possible to demonstrate seroconversion.

Repeat serology on 29 December 2022 (SO +19 weeks) demonstrated persistent IgM and IgG positivity. CK transiently increased to 340 U/L, with CRP 11 mg/L. Cardiac troponin remained within reference intervals throughout this period.

Bisoprolol and ramipril were commenced on 14 December 2022 but discontinued due to intolerance. Tachycardia improved without ongoing beta-blocker therapy.

### Pre-treatment study timepoint and treatment

On 1 February 2023 (SO + ~ 24 weeks), the first standardised Infectious Diseases–led longitudinal assessment was performed. This pre-treatment study timepoint (1 February 2023) served as the time zero analytical reference point for longitudinal modelling and biomarker analyses.

Intravenous ceftriaxone (2 g daily) was commenced immediately following blood sampling and continued for 28 days. Oral doxycycline (100 mg twice daily) began on 21 February 2023 and continued for 90 days.

At this timepoint, ANA had decreased to 1:40. RF ranged between 42 and 66 IU/mL. Anti-CCP antibodies were <1 U/mL on 24 September 2020 and again <1 U/mL on 22 August 2024.

Historical testing prior to symptom onset (24 September 2020) had already demonstrated elevated RF (28 IU/mL), confirming RF seropositivity that pre-dated the current illness.

### Follow-up and clinical outcome

Structured clinical evaluation included Horowitz Questionnaire Score (HQS) reflecting Multiple Systemic Infectious Disease Syndrome domains (24), Karnofsky Performance Status (KPS) (25), and blood sampling, followed immediately by initiation of intravenous ceftriaxone (Treatment Day 1).

Symptoms improved progressively. HQS decreased from 47 to 18 and KPS improved from 70 to 100 by Month 12.

Multisystem symptoms resolved in parallel with inflammatory marker normalisation and *Borrelia*-specific IgG contraction.

Intermittent, non-migratory arthralgia persisted with persistent rheumatoid factor (RF) seropositivity without clinical synovitis.

Follow-up TTE in mid-2024 demonstrated a mildly dilated left ventricle with low-normal systolic function, trivial atrioventricular valve regurgitation, and a patent foramen ovale, consistent with stable post-inflammatory remodelling.

At cardiology review on 13 June 2024, he was asymptomatic, medication-free, and reported no exertional limitation or palpitations.

The mature serologic and immune-mediator profile observed at the pre-treatment study timepoint was compatible with infection acquired during either European travel or following subsequent Sydney tick exposure and does not permit geographic attribution.

Overall findings were consistent with probable *Borrelia*-associated inflammatory cardiomyopathy in the absence of obstructive coronary disease.

**Figure 1:**
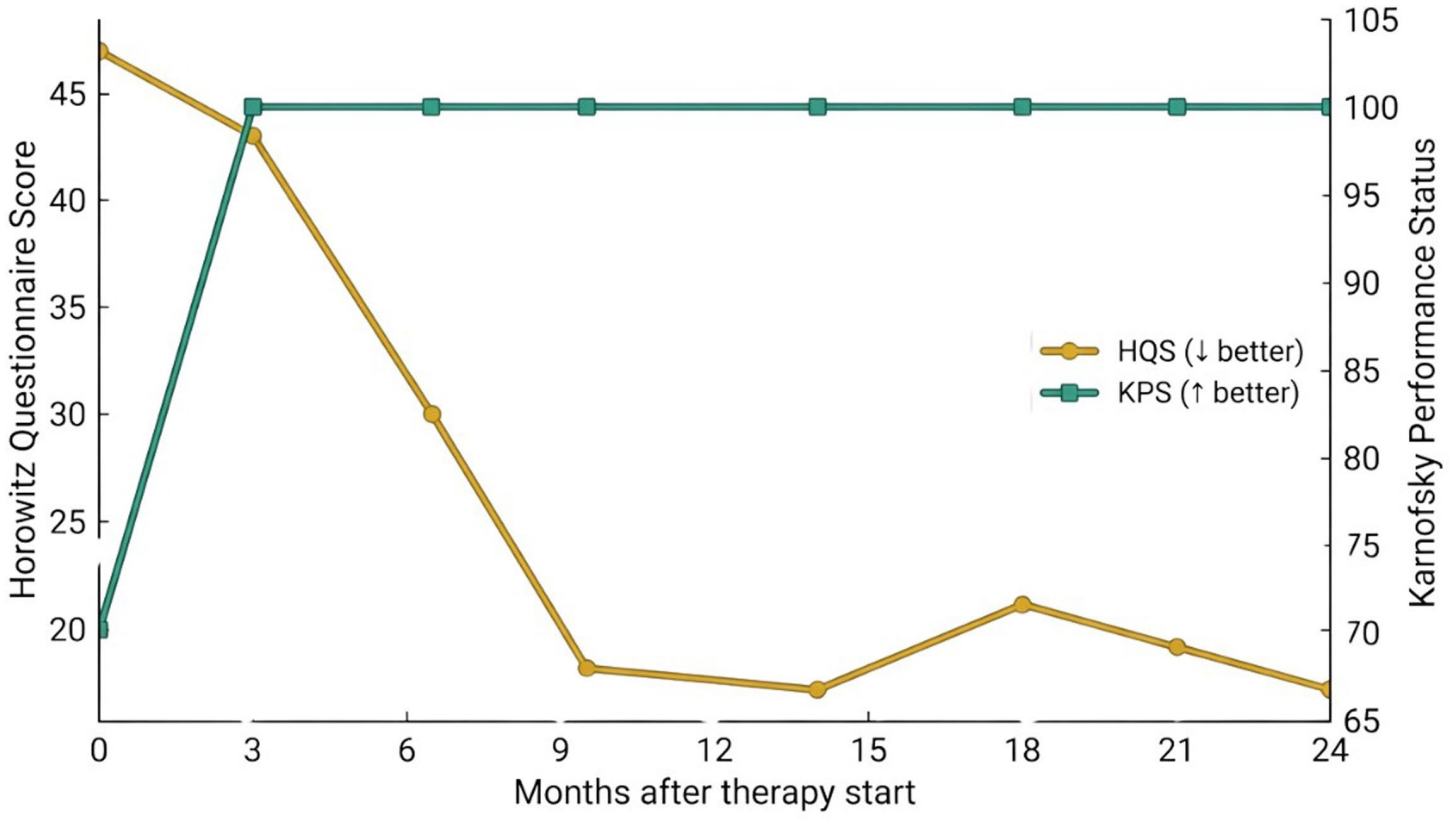
Therapy and functional outcomes over time. Timeline of antimicrobial therapy (intravenous ceftriaxone followed by oral doxycycline) alongside longitudinal functional assessments. Analytical Day 0 (1 February 2023; SO + ~ 24 weeks) is defined as the first pre-antimicrobial blood collection immediately preceding therapy initiation. Antimicrobial therapy commenced on the same calendar day and is designated as Day 1 of treatment. Treatment sequencing and cessation dates are detailed in Supplementary Table S1b. Symptom burden was tracked using the Horowitz Questionnaire Score (HQS), while global performance was measured using the Karnofsky Performance Status (KPS) scale (24, 25). Both metrics demonstrated progressive improvement from baseline through subsequent follow-up timepoints (Months 3, 4, 8, 12, and up to Month 24), correlating with clinical recovery and immune recalibration (see Figures 2, 3). Raw data are provided in Supplementary Table S4.

Key clinical and treatment milestones are summarised in Figure 1 and Supplementary Tables S1, S1b.

## Methods

### Study timepoints and clinical assessment

Symptom onset (SO; 16 August 2022) represents Clinical Day 0 of illness and is used for chronological reconstruction but not as the analytical reference for longitudinal modelling.

The pre-treatment study timepoint (1 February 2023) was designated Analytical Day 0 for modelling purposes and serves as the reference point for longitudinal clinical, serologic, cellular, and immune-biomarker analyses. This analytical reference is distinct from Clinical Day 0 (16 August 2022) and from the first serologic detection of IgG reactivity (18 November 2022; see Supplementary Table S1b).

Symptoms were assessed using the Horowitz Questionnaire Score (HQS) (Supplementary Table S5) (24), and functional status was evaluated using the Karnofsky Performance Status (KPS) scale (25).

### Pathology testing

Diagnostic testing was performed in NATA/RCPA-accredited ISO 15189 laboratories. Soluble immune-mediator assays were conducted at Eve Technologies (Calgary, Canada), a CLSI-validated reference laboratory using externally validated multiplex cytokine platforms.

### Microbiology, haematology and biochemistry

Routine testing included microbiological investigations, white cell counts (WCC), differential counts, inflammatory markers, cardiac biomarkers (cardiac troponin and creatine kinase), and autoantibody screening (ANA, RF, anti-CCP) (Supplementary Table S4).

### Serology

First-tier screening used the DiaSorin LIAISON^®^ XL *Borrelia* IgG/IgM chemiluminescence immunoassays. Where available, EUROIMMUN Anti-*Borrelia* ELISA (IgM/IgG; VlsE, p41, OspC) was also reviewed. Confirmatory testing employed the EUROIMMUN EUROLINE-RN-AT IgG line immunoblot, incorporating recombinant antigens from *Borrelia afzelii, B. burgdorferi,* and *B. garinii* (VlsE variants, p83, p58, p39, p41, OspC). Native *Borrelia* lysate control bands (LBa/LBb) were recorded for assay completeness and displayed for visual reference in figure representations only. These bands were excluded from diagnostic interpretation (Supplementary Table S1). IgG positivity followed EUCALB criteria (≥5 bands). Band interpretation used manufacturer criteria without densitometric quantification.

Co-infection serology with alternative tick-borne and systemic pathogens were excluded and are detailed in Supplementary Table S4.

### Molecular testing

Multiplex PCR (VIASURE BAC and Tick-Borne Diseases panels, Certest Biotec) targeted *Borrelia sensu lato* (s.l.), *B. miyamotoi*, *B. hermsii*, *Anaplasma phagocytophilum*, *Coxiella burnetii*, *Rickettsia* spp., *Babesia* spp., *Ehrlichias* spp., and tick-borne encephalitis (TBE) virus.

### Immune-mediator and biomarker analysis

Plasma cytokines, chemokines, and growth factors were quantified using the 71-plex Clinical platform (HD71-CLIN), integrating the MILLIPLEX® Human Cytokine 48-Plex (HCYTA-60 K) and 23-Plex (HCYP2MAB-62 K) assays run on Luminex 100/200™ instruments. Data were interpreted using the immune-cluster framework described by Polley et al. (11) to characterise inflammatory, chemotactic, angiogenic, and regulatory mediator patterns (Supplementary Tables S2, S3). All assays were performed by Eve Technologies (Canada).

### Therapeutic intervention and tolerability

Antimicrobial therapy commenced on the same calendar day following Analytical Day 0 sampling (Treatment Day 1). Treatment Day numbering refers to therapy duration and is independent of modelling timepoints.

Treatment commenced with intravenous ceftriaxone (2 g daily for 28 days; initiated 1 February 2023) followed by oral doxycycline (100 mg twice daily for 90 days; initiated on Treatment Day 21; 21 February 2023). Treatment was well tolerated, with only transient doxycycline-related nausea. No steroids, immunomodulators, or adjunctive therapies were used.

### Statistical analyses

Analyses were performed in R version 4.3.2 (R Foundation for Statistical Computing). Immune-mediator concentrations were log-transformed and summarised descriptively. Pearson correlations examined associations between immune-cluster patterns and clinical indices. Visualisation used ggplot2 and ComplexHeatmap (26).

## Results

### Clinical course

Prospective symptom burden and functional status commenced at Analytical Day 0 (1 February 2023; pre-antimicrobial therapy) using HQS and KPS. Prior symptoms were documented clinically but not retrospectively scored. Longitudinal HQS domain scores and KPS values are summarised in Supplementary Table S5, with chronological alignment of symptom assessment, serologic testing, and antimicrobial therapy shown in Figure 1 and detailed in Supplementary Tables S1, S1b. Symptoms improved rapidly following treatment (Figure 1), with the HQS decreasing from 47 to 18 and KPS improving from 70 to 100 by Month 12. Serial echocardiography remained stable throughout follow-up.

### Microbiology, serology and molecular testing

Serology demonstrated broad IgG reactivity across multiple diagnostic antigens, including VlsE variants, OspC, p39, and p58, consistent with disseminated *Borrelia* infection (Figure 3; Supplementary Table S1) (3, 6, 12, 14, 27–29).

IgM first detected on 18 November 2022 (SO +13 weeks) contracted progressively with complete seroreversion by Months 8–12.

Pre-treatment serology demonstrated a multi-antigen *Borrelia* IgG profile prior to antibiotic initiation (Supplementary Figure S3 and Supplementary Tables S1, S1b).

Longitudinal immunoblotting showed contraction toward persistent VlsE-dominant reactivity by Month 24, with waning *B. garinii*–associated bands (Figure 3).

Persistent p41 and native lysate bands (LBa/LBb) were recorded descriptively and excluded from diagnostic interpretation (Supplementary Table S1). Parallel IgG maturation, IgM resolution, and cytokine normalisation coincided with transition toward a vascular-repair immune signature, consistent with recognised patterns excluding isolated IgM persistence (30).

Whole-blood multiplex PCR and co-infection serology were negative (Supplementary Table S4). *Bartonella henselae* IgG seropositivity reflected prior exposure or cross-reactivity (31–33).

Although immunoblot patterns suggested European *Borrelia e*xposure, no isolate was obtained for genotyping. Stool multiplex PCR for pathogens and parasites was negative.

### Haematology and immunochemistry

Inflammatory markers demonstrated temporal variation across the illness course (Supplementary Table S4). High-sensitivity troponin remained <2 ng/L across serial measurements, arguing against necrotising myocardial injury and supporting a mild inflammatory cardiomyopathy phenotype. CRP peaked at 20.3 mg/L at SO +13 weeks and normalised following therapy. D-dimer measured on 21 October 2022 was mildly elevated at 0.59 mg/L, prompting evaluation for pulmonary embolism; computed tomography pulmonary angiography was negative. CK transiently increased to 340 U/L at SO +19 weeks before returning to reference intervals. Procalcitonin measured on 1 February 2023 (Analytical Day 0) was <0.02 μg/L, not suggestive of systemic bacterial sepsis. Serum angiotensin-converting enzyme (ACE) measured 24 U/L (22 August 2024), reducing the likelihood of sarcoid-associated cardiomyopathy in the differential diagnosis. ANA titres were low-level and decreased from 1:80 to 1:40; however, this represents a single doubling dilution and may fall within expected inter-assay variability, particularly as samples were not tested in parallel. The absence of anti-CCP seropositivity and clinical synovitis does not support rheumatoid arthritis or RA-associated inflammatory cardiomyopathy.

### White-cell count and immune-cell kinetics

White-cell dynamics mirrored soluble-mediator transitions, with mild early leukocytosis during innate/Th1 activation (B1/B3), followed by mid-course suppression during the B2/H stromal–vascular repair phase, and complete normalisation by Month 24 (Supplementary Table S4).

**Figure 2:**
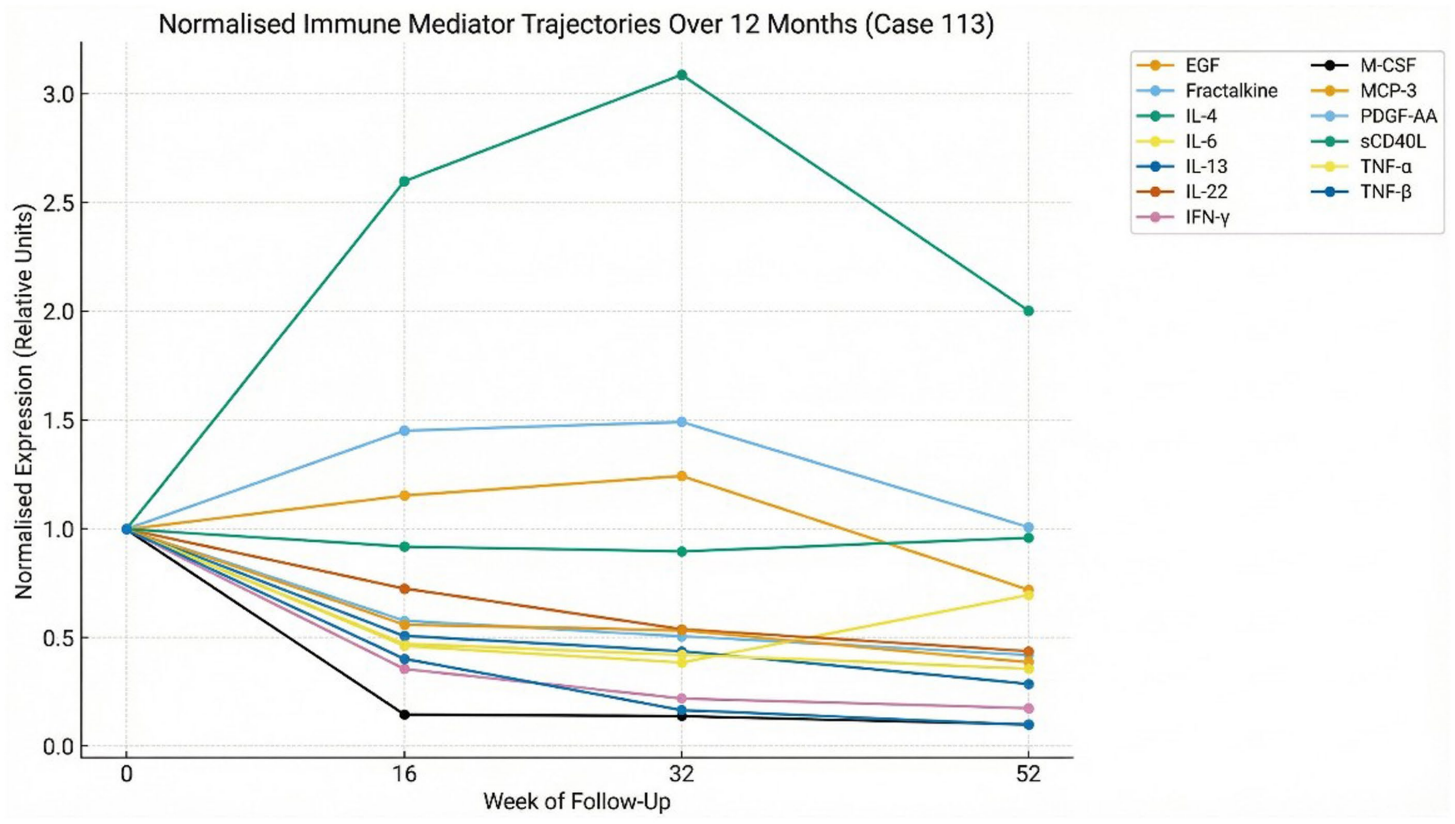
Longitudinal cytokine trajectories over 12 months (Case 113). Normalized soluble-mediator profiles demonstrate early mixed B1/B2 inflammatory–stromal activation at analytical day 0 (1 February 2023; SO +24 weeks; pre-antimicrobial therapy) (IL-6, TNF-*α*, IFN-*γ*, IL-4, IL-13, IL-22, fractalkine), followed by a dominant Group H vascular-repair phase led by PDGF-AA, EGF, and sCD40L during and following antimicrobial therapy. TNF-*β* (LT-α) showed early lymphoid activation with rapid down-regulation. By month 12, cytokine levels approached baseline, indicating immune resolution and endothelial–stromal stabilization. This immune trajectory paralleled clinical recovery and progressive serological contraction (Figure 3), consistent with functional immune-cluster transitions (Table 1; Supplementary Tables S2, S3).

Immunophenotyping showed low–low-normal CD19^+^ B-cell and CD16^+^CD56^+^ NK-cell counts consistent with early redistribution during inflammatory B1/B3 activation, with persistent low circulating levels during the B2/H repair phase (IL-4/IL-13/IL-22/sCD40L-driven), followed by return toward homeostasis by Months 12–24 (Figure 2).

**Figure 3:**
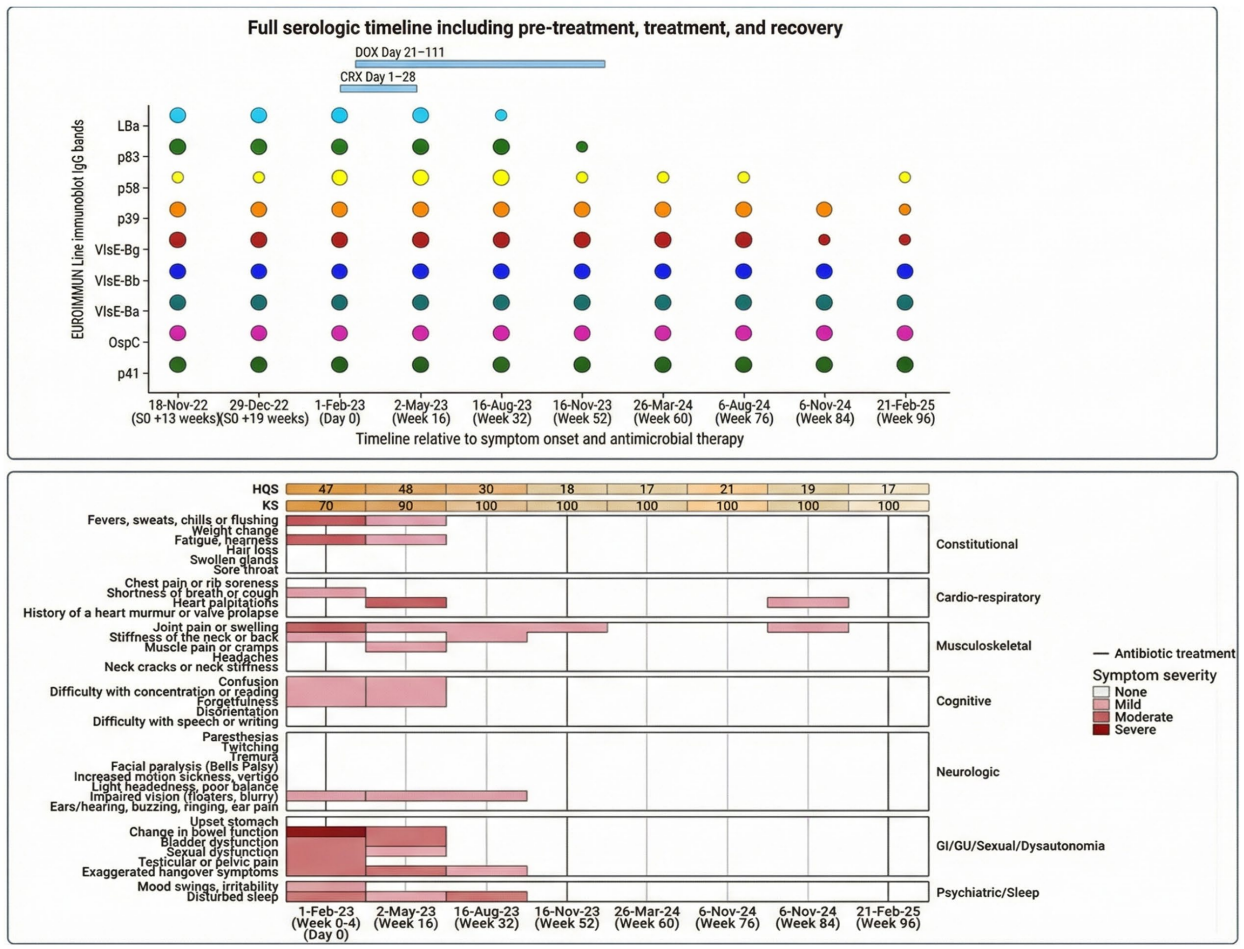
Progressive contraction in *Borrelia* IgG immunoblot reactivity across treatment phases and clinical recovery. For longitudinal modelling, baseline refers to the first standardised Infectious Diseases–led clinical and laboratory assessment performed on 1 February 2023 (SO +24 weeks), designated Analytical Day 0 and immediately preceding antimicrobial initiation (Treatment Day 1). This analytical baseline is distinct from the first reactive immunoblot obtained on 18 November 2022 (SO +13 weeks; Day 95; Supplementary Table S1b). Ceftriaxone was initiated immediately after Analytical Day 0 sampling (Treatment Day 1) and administered for 28 days; oral doxycycline commenced on Treatment Day 21 and continued for 90 days (Treatment Day 111) (Supplementary Table S1b). Longitudinal immunoblot evolution from the first reactive serum (18 November 2022; SO +13 weeks; Day 95) through Month 24 demonstrates progressive qualitative contraction of IgG reactivity from a broad nine-band profile to persistent residual VlsE-dominant reactivity, with waning of *B. garinii*–associated VlsE bands by Months 18–24 (Supplementary Table S1). Coloured markers represent recombinant *Borrelia* antigens (p41, OspC, VlsE variants, p39, p58, p83). Persistent p41 reactivity likely reflects immune memory and was not considered diagnostically significant. Native lysate control bands (LBa) are shown for visual reference only and were excluded from longitudinal interpretation (Supplementary Table S1). Dots indicate presence of IgG reactivity; paler dots denote weaker or resolving reactivity. Band strength classification followed manufacturer visual interpretation criteria rather than densitometric quantification. Serologic contraction occurred in parallel with improvement in HQS and KPS/KS (Supplementary Tables S1, S5). The horizontal gradient bar denotes clinical phases, and the vertical line indicates antibiotic initiation. Follow-up timepoints are presented as categorical intervals and do not represent proportional elapsed time. This figure illustrates longitudinal serologic evolution relative to treatment and clinical recovery and does not infer timing or geographic origin of infection.

### Soluble immune-mediator profiling

Longitudinal profiling revealed a structured, phase-specific trajectory (Table 1; Supplementary Tables S2, S3; Supplementary Figures S1, S2).

**Table 1:** Cytokine trajectories, functional grouping, and phase-specific immunologic interpretation.

Timepoint relative to analytical day 0 (1 February 2023)	Dominant immune markers elevated	Functional cluster (11)	Biological significance	Interpretation
Analytical Day 0 (1 February 2023; pre-antimicrobial; SO + ~ 24 weeks)	Elevated EGF, Fractalkine, IL-4, IL-13, IL-22, M-CSF, MCP-3, PDGF-AA, TNF-β ± IL-1β, IL-6, IFN-γ	B1/B2 predominant with B3 contribution and early Group H engagement	Mixed innate (IL-1β, IL-6, IFN-γ) and stromal/Th2–Th22 signalling; endothelial activation (fractalkine)	Residual inflammatory–stromal activation with stromal/endothelial involvement following symptom onset (SO); consistent with early innate + adaptive co-activation described in Results
Month 4 (Week 16)	Rising PDGF-AA, EGF, sCD40L; persistent IL-4; falling fractalkine; TNF-β normalising	Transition B1/B2 → H (Initiation of Vascular Repair)	Down-regulation of innate inflammation; rise in angiogenic and platelet-derived mediators	Controlled resolution phase: innate activation contracting while vascular-repair signalling expands. Mirrors cytokine-defined shift described in Figure 2
Month 8 (Week 32)	Sustained PDGF-AA, EGF, sCD40L; IL-4 stable; fractalkine and TNF-β within range; Th17 axis undetectable	Group H – Vascular Remodelling / Stromal Repair	Endothelial and stromal recovery; no detectable Th17-associated cytokine elevations (IL-17A/F, IL-23)	Sustained vascular integrity and immune quiescence; immune trajectory fully shifted toward platelet–stromal repair, consistent with B-cell/NK-cell redistribution patterns
Month 12 (Week 52)	PDGF-AA, EGF, sCD40L trending toward normal; IL-4 mildly elevated; fractalkine stable; TNF-β low-normal	Group H – Stable Repair and Immune Homeostasis	Mature endothelial–stromal signalling; low-grade Th2-linked activity	Durable immune resolution with no chronic inflammatory or Th17-driven activity; aligns with clinical recovery and serologic contraction

Phase-specific cytokine trajectories demonstrating the coordinated transition from mixed B1/B2 inflammatory–stromal activation with B3 innate contribution to Group H vascular-repair programming over 12 months. At Analytical Day 0 (1 February 2023; first standardised pre-antimicrobial sampling timepoint, approximately 24 weeks post–symptom onset), cytokines showed mixed innate (IL-1β, IL-6, TNF-α, IFN-γ), stromal/Th2–Th22 (IL-4, IL-13, IL-22), and endothelial activation (fractalkine), with early engagement of repair mediators (PDGF-AA, EGF). By Month 4, declining innate cytokines with rising PDGF-AA, EGF, and sCD40L indicated a B1/B2 → H transition. By Months 8–12, cytokine patterns demonstrated predominance of a Group H repair profile dominated by PDGF-AA, EGF, and sCD40L, with persistent low-grade IL-4–associated stromal repair signalling. These trajectories correspond to the immune-mediator patterns described in the Results (“Soluble Immune-Mediator Profiling”) and are supported by lymphocyte-subset trends and serologic contraction. Cytokine groupings follow Polley et al. (11), with quantitative values provided in Supplementary Tables S2–S4. Timepoints are expressed relative to the pre-antimicrobial baseline (Analytical Day 0); a consolidated calendar timeline of clinical events, serologic testing, and treatment initiation is provided in Figure 1 and Supplementary Table S1b.

At Analytical Day 0, elevations reflected mixed T-helper type 2 and T-helper type 22 (Th2/Th22) stromal–endothelial activation with transient Th1 and early lymphoid involvement (B1/B2) (11, 34). This phase was characterised by coordinated innate, stromal, and endothelial activation involving interleukins (IL-1*β*, IL-6, IL-4, IL-13, IL-22), tumour necrosis factor-*α*/β (TNF-α/β), interferon-*γ* (IFN-γ), epidermal growth factor (EGF), platelet-derived growth factor-AA (PDGF-AA), and fractalkine (CX3CL1).

By Months 8–12, mediator patterns converged toward a Group H vascular-repair phenotype dominated by PDGF-AA, EGF, sCD40L, IL-10, and IL-1RA, consistent with inflammatory resolution (Figure 2) (11).

Th17-associated cytokines (IL-17A, IL-17F, IL-23) remained undetectable, distinguishing this trajectory from Th17-skewed immune profiles reported in antibiotic-refractory Lyme disease and Post-Treatment Lyme Disease Syndrome (PTLDS), where persistent immune activation and dysregulated host responses have been described (8, 35). Similar chronic symptom complexes, such as DSCATT, may reflect overlapping but heterogeneous immune mechanisms (6).

Peripheral cellular kinetics paralleled soluble-mediator dynamics, with redistribution of CD19^+^ B cells and CD16^+^CD56^+^ NK cells during early B1/B3 activation, followed by sustained low circulating levels during the B2/H repair phase. White-cell dynamics followed this pattern, with mild leukocytosis early, mid-phase suppression during stromal–vascular repair, and normalisation by Month 24.

By Months 12–24, B-cell and NK-cell subsets returned to low-normal homeostasis, consistent with restoration of vascular–stromal equilibrium. This integrated cytokine–cellular trajectory aligned clinical recovery with serologic contraction and immune resolution.

### Functional outcomes

Improvements in validated systemic, neurological, musculoskeletal, cognitive, and autonomic domains paralleled immune normalisation and *Borrelia*-specific IgG contraction, consistent with reversible inflammatory cardiomyopathy.

## Discussion

This report does not establish endemic *Borrelia* transmission in Australia but illustrates the clinical, serologic, and immunologic features consistent with probable *Borrelia*-associated cardiomyopathy. It should not be interpreted as evidence of local transmission or epidemiologic inference regarding endemicity.

Cardiac manifestations of LB are rare but recognised (12, 13, 15–19). While myocarditis is a term commonly used in Lyme carditis literature, inflammatory cardiomyopathy more precisely reflects the imaging and biomarker findings in this case.

Two-tier serology, mild global cardiomyopathy, LBBB, and normal coronary arteries were consistent with early disseminated infection (13, 16, 17), aligning with reversible conduction abnormalities and transient ventricular dysfunction described in Lyme carditis (18, 20, 36). High-sensitivity troponin remained within reference intervals, supporting mild inflammatory cardiomyopathy rather than necrotising myocardial injury (16, 17).

COVID-19 vaccine–associated myocarditis was considered unlikely given persistently normal troponin, stable echocardiographic findings, a benign trajectory, and a discordant immune-mediator profile (10, 21–23). Viral, sarcoid (ACE 24 U/L), and autoimmune causes were excluded (14, 16–20). RF seropositivity pre-dated illness, anti-CCP antibodies remained negative, and no synovitis developed (37), arguing against rheumatoid arthritis or RA-associated inflammatory cardiomyopathy (9).

The IgG immunoblot profile fulfilled international criteria for early disseminated infection. VlsE (*B. afzelii, B. burgdorferi, B. garinii*) reactivity remained detectable longitudinally, with relative attenuation of the *B. garinii* component consistent with late-phase resolved infection patterns. Reactivity to conserved antigens (p58, p83) was interpreted descriptively and not used for genospecies attribution (38–43).

Longitudinal IgG contraction with IgM seroreversion demonstrated serologic resolution (30, 44–49), although interpretation of immunoblot patterns remains constrained by recognised cross-reactivity and qualitative assessment within established criteria (30).

Whole-blood PCR is recognised to have low sensitivity in disseminated infection due to transient, low-level spirochaetaemia (50, 51).

Immune profiling demonstrated a structured transition from inflammatory signalling at the pre-treatment study timepoint to a Group H vascular-repair phenotype characterised by PDGF-AA, EGF, and sCD40L, consistent with established frameworks (11) (Supplementary Tables S2, S3). Cellular kinetics showed early innate/Th1-associated redistribution (B1/B3) followed by quiescent B2/H stromal–vascular repair paralleling clinical recovery. Th17-associated cytokines remained undetectable, distinguishing this pattern from antibiotic-refractory Lyme arthritis, PTLDS, and DSCATT profiles (8, 11, 35). Although similar reparative immune-signature patterns have been described in resolving Lyme borreliosis and other post-infectious states (11, 35), persistent immune activation in PTLDS and DSCATT has in some studies been linked to proposed ongoing antigenic or immune stimulation, including responses to *Borrelia* components or tick-derived proteins (52–57). Application of functional cytokine clustering remains exploratory and supports biological interpretation rather than mechanistic causation.

Symptom improvement tracked with HQS and MSIDS domains (Supplementary Table S5 and Supplementary Figure S4), paralleled IgG contraction and immune-mediator normalisation. Overlap with symptom complexes described in ME/CFS, PTLDS, and PVD (9, 10) was noted, yet resolution alongside normalisation supported a reversible post-infectious inflammatory process rather than chronic multisystem illness. HQS, while partially validated and distinct from instruments such as GSQ-30 (24, 58), provided structured longitudinal assessment.

Early antimicrobial therapy has been associated with favourable cardiac outcomes in disseminated LB (3, 6, 12, 14, 16–20, 27–29, 36, 59–62), though causality cannot be inferred from a single case.

Strengths of this case report include longitudinal serology integrated with serial immune-signature profiling and cellular kinetics despite absence of pathogen isolation. Concordance between clinical recovery, serologic maturation and immune-mediator normalisation supports resolution.

Limitations include inability to isolate the organism, preventing genotyping, and uncertainty regarding geographic acquisition. The mature immunologic profile at first detection (38, 39, 41, 47) is compatible with infection acquired in Europe or following subsequent Sydney exposure and does not distinguish between these possibilities.

These findings support translational research and surveillance in Australian TBD settings (11, 41–43, 47), complementing pathogen surveillance efforts (2, 4) and DSCATT research frameworks (2, 4, 7, 42, 45, 55–57, 63–65).

Overall, this case demonstrates the value of integrating serologic and immune-signature profiling in complex tick-associated presentations, within the limitations of single-case observational inference.

## Conclusion

This case demonstrates complete clinical, serologic, and immunologic recovery from probable *Borrelia*-associated myocarditis following antibiotic therapy. Serologic contraction and transition to a Group H vascular-repair immune signature paralleled complete clinical recovery. No PTLDS–like phenotype was observed during follow-up. Integrated serology and immune-signature profiling may improve diagnostic confidence in settings lacking locally validated assays and support clearer characterisation of “Lyme-like” illness and DSCATT in Australia.

This case report adheres to the CARE Checklist (Supplementary Table S6).

## Data Availability

The original contributions presented in the study are included in the article/Supplementary material, further inquiries can be directed to the corresponding author.
