# Supplementary material for: Probable European-profile Borrelia-associated myocarditis in an Australian patient with immune resolution following early therapy: a case report
Source: Front Med (Lausanne). 2026 Apr 15;13:1784007. doi: 10.3389/fmed.2026.1784007 (PMC13126735; doi:10.3389/fmed.2026.1784007)
Supplement: Supplementary file 1 [file Data_Sheet_1.pdf]

Title: Probable European-Profile *Borrelia*-Associated Myocarditis in an Australian Patient With Immune Resolution Following Early Therapy: A Case Report

Katerina Mitsakos[2,5](ORCID), Pamela E. Heuer[2], Tiziana Beninati[2], Daniel J. Polley[4], Richard J. Schloeffel[3], and Bernard J. Hudson[1,5]* (ORCID).

Supplementary Material

Supplementary (S) Figures (S1–S4) are provided in the accompanying Word/PDF file *CaseReport_Borreliosis_Myocarditis_EarlyTherapy_Australia_SupplementaryFigures (Figures S1–S4).docx*, which includes the following:

- **Figure S1.** Mean $\Delta\log_{10}$ Change (Record 113) by Functional Cytokine–Chemokine Group (Polley et al., 2023) derived from Supplementary Table S3
- **Figure S2.** Full $\Delta\log_{10}$ cytokine trajectories (71 analytes) grouped by functional category (Polley et al., 2023).
- **Figure S3.** Full serologic timeline including pre-treatment samples relative to symptom onset (SO), treatment initiation, and longitudinal recovery.
- **Figure S4.** Longitudinal symptom severity profile across clinical domains relative to treatment and follow-up.

Cross-references to these supplementary files appear throughout the manuscript where relevant (e.g., see Supplementary Table S2; Supplementary Figure S1).

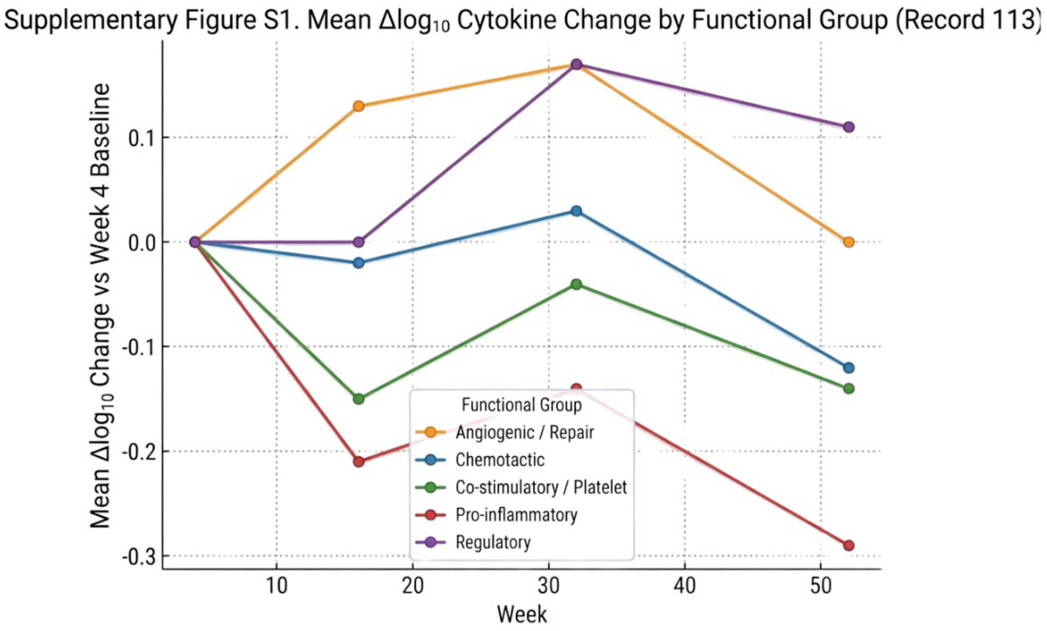

Figure S1. Mean $\Delta\log_{10}$ Change (Record 113) by Functional Cytokine–Chemokine Group (Polley et al., 2023) derived from Supplementary Table S3

Figure S1 visualises the longitudinal immune trajectory from Analytical Day 0 (1 February 2023; pre-antimicrobial baseline) to Week 16 to Week 32 to Week 52, showing angiogenic/repair mediators (orange cluster) peaking at Week 32 and then normalising; chemotactic (blue cluster) and co-stimulatory/platelet groups (green cluster) trending downward; pro-inflammatory cytokines (red cluster) steadily declining; and regulatory mediators (purple cluster) rising through mid-follow-up, a coordinated shift from early immune activation to vascular repair and regulatory predominance by 12 months.

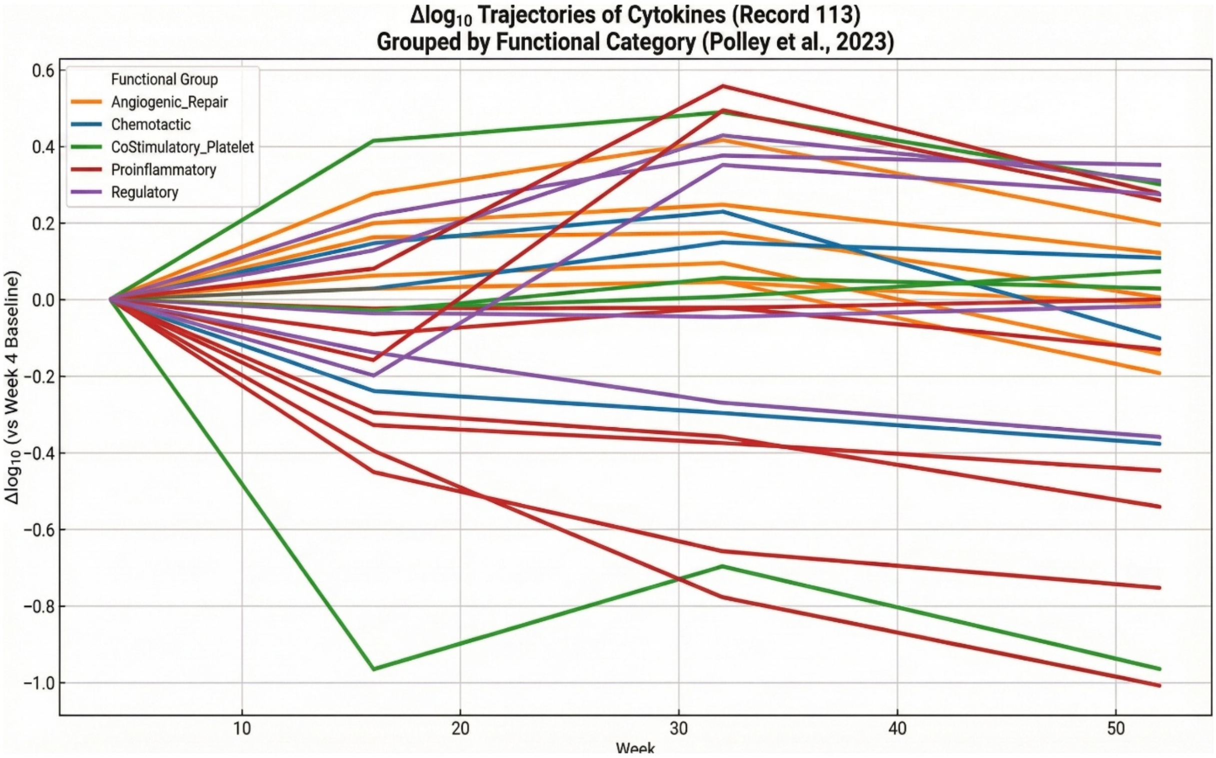

Figure S2. Full $\Delta\log_{10}$ cytokine trajectories (71 analytes) grouped by functional category (Polley et al., 2023).

Each thin line represents one cytokine's $\Delta\log_{10}$ change relative to the Analytical Day 0 (1 February 2023; pre-antimicrobial baseline), coloured by functional cluster. Bold overlay lines indicate group means ± SE. The figure visualises heterogeneous but coordinated immune trajectories across 71 analytes, demonstrating transition from early activation to vascular repair and regulatory equilibrium by Week 52. Underlying cytokine concentrations, directional trends, and functional interpretations are provided in Supplementary Table S2, from which $\Delta\log_{10}$ trajectories were derived.

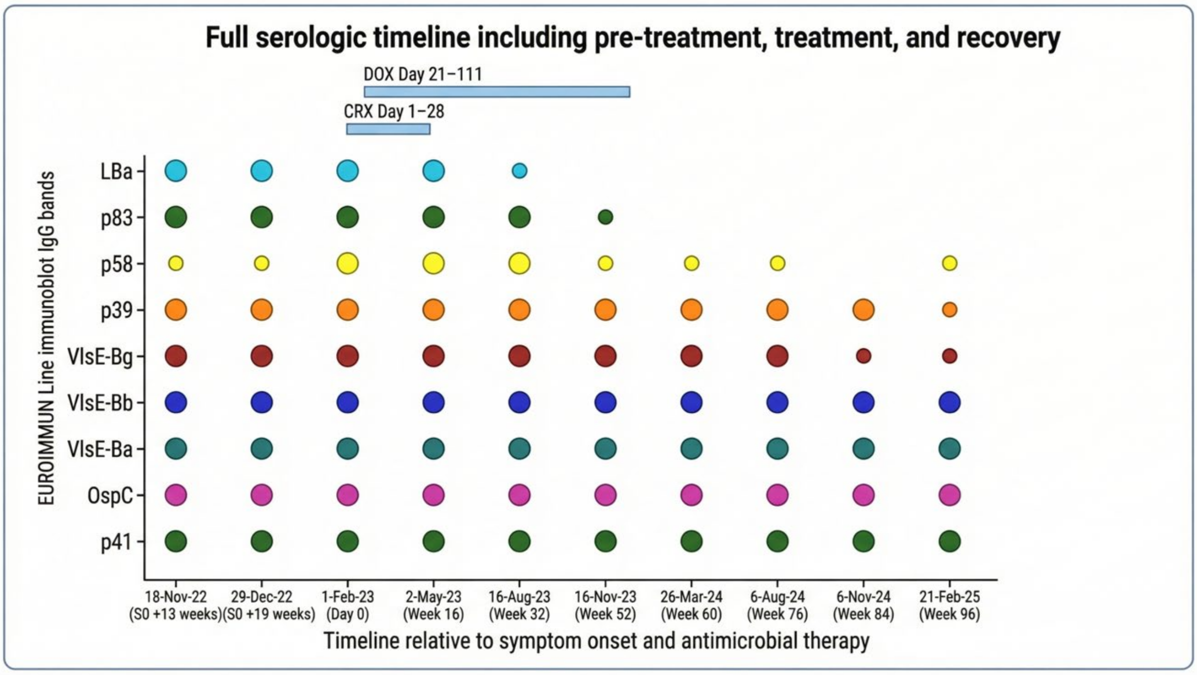

Figure S3. Full serologic timeline including pre-treatment samples relative to symptom onset (SO), treatment initiation, and longitudinal recovery.

Longitudinal serologic profile showing IgG reactivity to recombinant *Borrelia* antigens across pre-treatment, treatment, and post-treatment recovery. The x-axis is anchored to symptom onset (SO; 16 August 2022), calendar-dated pre-treatment samples, and follow-up timepoints through Week 96. Coloured markers represent recombinant antigens (p41, OspC, VlsE [*B. afzelii, B. burgdorferi, B. garinii*], p39, p58, p83); dots indicate presence of IgG reactivity, with paler dots denoting weaker or resolving reactivity (intensity not quantitatively scaled; band strength classification followed manufacturer visual interpretation criteria). Native Borrelia lysate control bands (LBa) are shown for visual reference only and were excluded from longitudinal interpretation. Persistent p41 reactivity likely reflects immune memory and was not considered diagnostically significant.

Clinical Day 0 corresponds to symptom onset (16 August 2022), whereas Analytical Day 0 (1 February 2023) represents the pre-antimicrobial baseline blood draw used for longitudinal analyses. Ceftriaxone (CRX) was initiated immediately after Analytical Day 0 sampling (Treatment Day 1) and administered for 28 days; oral doxycycline (DXC) commenced on Treatment Day 21 as overlapping sequential therapy and continued for 90 days, completing on Treatment Day 111 (treatment bars shown in therapy days only). This figure contextualises multispecies IgG positivity established prior to treatment and the subsequent contraction of serologic reactivity during recovery; detailed band summaries and diagnostic interpretation are provided in Supplementary Tables S1 and S1b.

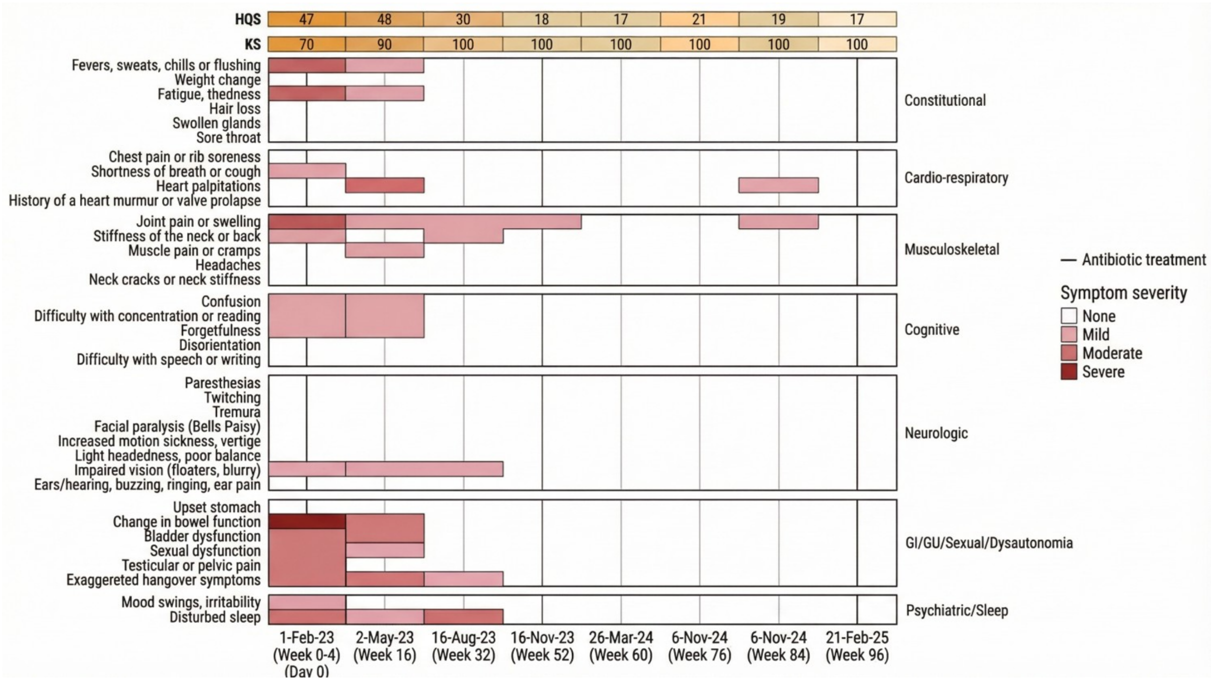

Supplementary Figure S4. Longitudinal symptom severity profile across clinical domains relative to treatment and follow-up.

Symptom severity was assessed using an adapted HQS-derived framework and categorised as none, mild, moderate, or severe. Vertical reference line indicates initiation of antibiotic therapy. Domains are grouped by clinical system (constitutional, cardio-respiratory, musculoskeletal, cognitive, neurologic, gastrointestinal/genitourinary/dysautonomia, and psychiatric/sleep). HQS and KPS scores are shown longitudinally.

Interpretation of symptom domains should consider the validation statement provided in Supplementary Table S5.
